# Formation of Local Societies

**Published:** 1861-04

**Authors:** J. W. Van Osten, W. W. Allport, W. Muir Rodgers

**Affiliations:** Philadelphia; Chicago, Ill.; Shelbyville, Ky.


### FORMATION OF LOCAL SOCIETIES.

At the meeting of the American Dental Association, held at Washington, D. C., July, 1860, the undersigned were appointed a committee to confer with dental practitioners throughout the Union, with a view to the formation of local societies.

In the discharge of this duty, the most feasible plan that suggests itself to us for laying the matter before the profession is to take advantage of the facilities afforded by the dental journals.

We would respectfully suggest it to be a self-evident fact, recognized by every reflecting mind, that innumerable advantages must accrue to the individual members, as well as to the profession at large, by associated effort. Nothing is better calculated to break down local jealousies; to establish in their place agreeable and fraternal relations; to foster a desire for individual improvement; and beget that *esprit de corps*, which, wherever it exists, has a tendency to elevate the character and standing of any profession. Taking this broad view of the matter, it is deeply to be regretted that our profession should not have had its local societies established long since in every State and town of any size in the Union.

It is, however, gratifying to observe (and we take pleasure in recording the fact) that within the last year and a half a number of local associations have been formed, and we have reason to believe that the movement in favor of a national delegated association has had much to do with the organization of these societies. We trust their example will be followed by the profession in other sections of the country.

Having been appointed merely to confer with the profession on this subject, and as we have directed attention briefly to the advantages of associations, we do not feel it necessary to offer any farther suggestions. Trusting that the profession will feel the importance of acting promptly in this matter, we shall be happy to afford any information and assistance in our power that may be desired.

J. W. Van Osten, Philadelphia,<br>

W. W. Allport, Chicago, Ill.,<br>

W. Muir Rodgers, Shelbyville, Ky., } *Com.*

